# Aneuploidy screening of embryonic stem cell clones by metaphase karyotyping and droplet digital polymerase chain reaction

**DOI:** 10.1186/s12860-016-0108-6

**Published:** 2016-08-05

**Authors:** Gemma F. Codner, Loic Lindner, Adam Caulder, Marie Wattenhofer-Donzé, Adam Radage, Annelyse Mertz, Benjamin Eisenmann, Joffrey Mianné, Edward P. Evans, Colin V. Beechey, Martin D. Fray, Marie-Christine Birling, Yann Hérault, Guillaume Pavlovic, Lydia Teboul

**Affiliations:** 1 The Mary Lyon Centre, Medical Research Council Harwell Institute, Harwell Science and Innovation Campus, Didcot, OX11 0RD Oxon UK; 2 PHENOMIN, Institut Clinique de la Souris, ICS; CNRS, INSERM, Université de Strasbourg, Illkirch-Graffenstaden, Strasbourg, 67404 France

**Keywords:** Aneuploidy, Karyotype, Droplet digital PCR, Cell culture, Chromosome number, Multiplex assay, Embryonic stem cells

## Abstract

**Background:**

Karyotypic integrity is essential for the successful germline transmission of alleles mutated in embryonic stem (ES) cells. Classical methods for the identification of aneuploidy involve cytological analyses that are both time consuming and require rare expertise to identify mouse chromosomes.

**Results:**

As part of the International Mouse Phenotyping Consortium, we gathered data from over 1,500 ES cell clones and found that the germline transmission (GLT) efficiency of clones is compromised when over 50 % of cells harbour chromosome number abnormalities. In JM8 cells, chromosomes 1, 8, 11 or Y displayed copy number variation most frequently, whilst the remainder generally remain unchanged. We developed protocols employing droplet digital polymerase chain reaction (ddPCR) to accurately quantify the copy number of these four chromosomes, allowing efficient triage of ES clones prior to microinjection. We verified that assessments of aneuploidy, and thus decisions regarding the suitability of clones for microinjection, were concordant between classical cytological and ddPCR-based methods. Finally, we improved the method to include assay multiplexing so that two unstable chromosomes are counted simultaneously (and independently) in one reaction, to enhance throughput and further reduce the cost.

**Conclusion:**

We validated a PCR-based method as an alternative to classical karyotype analysis. This technique enables laboratories that are non-specialist, or work with large numbers of clones, to precisely screen ES cells for the most common aneuploidies prior to microinjection to ensure the highest level of germline transmission potential. The application of this method allows early exclusion of aneuploid ES cell clones in the ES cell to mouse conversion process, thus improving the chances of obtaining germline transmission and reducing the number of animals used in failed microinjection attempts. This method can be applied to any other experiments that require accurate analysis of the genome for copy number variation (CNV).

**Electronic supplementary material:**

The online version of this article (doi:10.1186/s12860-016-0108-6) contains supplementary material, which is available to authorized users.

## Background

Genome sequence data and the subsequent generation of targeted mutation libraries in mouse Embryonic Stem (ES) cells have facilitated the systematic analysis of gene function in mutant animal models [1]. Initial large-scale projects created over 1,300 mouse lines, annotated the function of over 800 mouse genes and piloted such intricate analyses in a high-throughput fashion [2, 3]. The remit of the International Mouse Phenotyping Consortium (IMPC) is to capitalise further on these resources and generate, characterise and disseminate up to 20,000 knock-out mouse lines [4]. Both the PHENOMIN Institut Clinique de la Souris (ICS) and the Mary Lyon Centre (MLC) at the Medical Research Council (MRC) Harwell are members of this worldwide coordinated consortium. Collectively, these two centres have so far imported and checked the karyotype of over 3,500 ES cell clones, by either cytological or ddPCR-based methods, for the high-throughput conversion of cells into mouse models. In this pipeline setting, the nature and scale of which is unusual in academia, both centres injected a large number of clones under standardised conditions, including the number of embryo hosts used, and each clone was generally not re-injected. The efficiency of the ES cell to mouse conversion process is essential to the success of such a programme. The consortium continually strives for improvements in germline transmission (GLT) efficiency, and the scale of the effort creates the opportunity to thoroughly test and assess improvements to this process. In doing so, we have developed and implemented new protocols to aid conversion from ES cell to mouse, one of which is described here.

Published data indicate that karyotypic instability of modified ES cells is a major reason for the failure of GLT [5–8]. It is widely accepted that chromosome abnormalities are frequently found in ES cell lines subjected to extended passages in culture [9–12]. Typically, mouse ES cell line abnormalities are a gain of Chr 8 and/or 11, and often loss of Chr Y, but each parental cell line may also show trends for other specific chromosomes anomalies (e.g J1 mESCs exhibit gain of chromosome 8 and structural rearrangements/Roberstonian translocations involving chromosome 11) [9–12]. Compound trisomy 8 and 11 can be observed in mouse ES cells, and frequency increases with passage of cells but does not seem to impact the ability of the cells to differentiate [10]. Trisomy 8 was shown to impact the GLT potential of ES cells [5], supporting the notion that karyotypic changes are a major reason for the lack of contribution from individual ES cell clones to the germline of chimeras.

At present, although Chr 8, 11 and Y have been highlighted as particularly unstable in cultured ES cells [9–12], no large-scale study has been performed to identify the chromosomes unstable at high frequency in the cell lines employed for the generation of gene targeting libraries derived from the C57BL/6N genetic background. Cotton et al. [13] proposed 50 % euploidy as the threshold at which clones are deemed acceptable for injection, as the 8 clones in their study with less than 50 % euploid cells did not yield GLT. However, no systematic study performed on a large number of clones describes the relationship between the percentage of aneuploid cells present in injected clones and their ability to transmit through the germline of founder animals.

Classically, analysis of Giemsa banded (G-banded) metaphase spreads, or other chromosome spread preparation-based methods are employed to identify aneuploidies [14]. We have published the description of the reference ideogram for mouse chromosomes by such methods [15]. Detailed karyotyping methods require extensive training and expertise which are impractical, expensive and time consuming in a high-throughput setting [16]. Here, we present a systematic analysis of chromosome anomalies in large cohorts of cultured ES cell clones of C57BL/6N and other genetic backgrounds. We document the relationship between the incidence of chromosome anomalies and GLT capacity, confirming the 50 % threshold of euploid cell contribution for efficient ES cell to mouse conversion. We identify four principal chromosomes involved, either singly or in combination, in all of the cases of aneuploidy in 138 C57BL/6N clones that we surveyed. We extend the study to include other parental ES cell lines and show different trends in terms of chromosome stability. We tease out the relationship between chromosome numbers and GLT potential of clones. Finally, we present a new, simple and affordable protocol, based on droplet digital polymerase chain reaction (ddPCR), which enables the rapid screening of clones grown in a minimum of a 96-well plate format. Whilst the protocol does not constitute a survey of the whole genome, it is sufficient to identify the clones most likely to support GLT and that are therefore worthy of microinjection.

## Results and discussion

### Chromosome number and GLT capacity

**Fig. 1:**
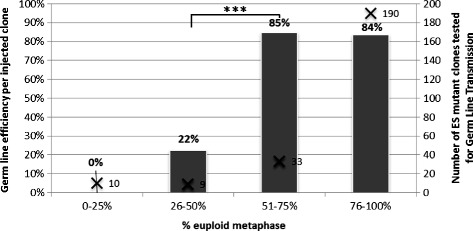
Contribution of euploid cells and GLT rate in all C57BL/6N-derived clones. Percentage of euploid metaphases observed by Giemsa staining metaphase spread-based karyotyping was compared to germ line efficiency obtained at ICS (left axis, grey). Number of ES mutant clones tested is indicated on the right axis (cross). Data was analysed using the Fisher Exact test and yielded a P value of 0.000806. False discovery rate (Q) calculated by the Benjamini-Hochberg procedure was 0.003224. This showed that clones with greater than 50 % euploid representation are preferable candidates for microinjection

As part of the IMPC programme [4], we microinjected large numbers of imported ES cell clones, many of them derived from the JM8 parental line [17], into blastocysts under standardised conditions. The aim of these microinjections is to create chimeric founders that will be mated to obtain GLT of the mutation targeted in the ES cells. Figure 1 presents the percentage of successful GLT per ES clone in relation to the percentage of euploid cells present. The micro-injected clones represent a large population, unbiased for gene families or pathways targeted, thus supporting the systematic analysis of the relationship between GLT potential and chromosome number. The dataset showed that clones with lower percentage of euploid cells have significantly diminished ability to contribute to the germline. The threshold at which GLT efficiency drops from over 80 % to just over 20 % is less than 50 % euploid metaphases. This reaffirms the conclusion that the threshold for efficient GLT is 50 % as proposed by Cotton and colleagues [13], and also suggests that this threshold is not specific to our culture conditions and processes.

Clones with less than 50 % of euploid cells, and thus with a low GLT efficiency, represent nearly 25 % of total clones (C57BL/6N background) that were generated [18]. To achieve efficient ES cell to mouse conversion, the accurate identification and removal of clones with abnormal chromosome numbers from the pipeline is clearly essential. If only clones with poor euploid contribution are available, successful GLT is likely to require the microinjection of larger numbers of blastocysts. Furthermore, our data suggest that, should GLT fail with a clone with a low percentage of euploid cells, sub-cloning and selection of karyotypically normal sub-clones, using the protocol presented here as a screening method, may rescue these ES cell lines and allow GLT.

In order to run an efficient ES cell to mouse conversion process, clones containing less than 50 % of euploid cells should not be injected when other clones carrying the same gene targeting event and a higher euploid complement are available. We extended our analysis to more than 1,500 different ES mutant clones of 129S2 or C57BL/6N genetic backgrounds and showed that the GLT capacity is directly correlated to the percentage of euploid metaphases detected by chromosome counting after Giemsa staining (Additional file 1: Figure S1). The data illustrate that parental clones and genetic background influence the likelihood of microinjection yielding GLT (ie. 22 % of the sub-clones of the P1 line, isolated from embryos of the 129S1 genetic background, with poor karyotype retain capacity to yield GLT in contrast with other lines isolated from more challenging genetic backgrounds). However, the 50 % threshold remains relevant to all parental ES cell lines, across the different genetic backgrounds included in our study, to identify the clones with higher GLT potential for microinjection.

For clones imported from large-scale collections [18], we analyzed both quality control pass and GLT rates of clones according to the source (listed in methods). We found that the identity of the distributor did not influence the frequency of chromosome number variation and/or overall GLT rates (Additional file 2: Figure S2), suggesting that the chromosome number instability we show here is more intrinsic to the JM8 C57BL/6N cell line used, than a consequence of the cell culture conditions applied prior to distribution.

### Unstable chromosomes in cultured ES cells

Identifying which chromosomes are prone to aneuploidy in C57BL/6N derived ES cells was integral to establishing the ddPCR karyotype screen. Additional copies of Chr 8 and/or 11, or loss of Chr Y are frequently observed in mouse ES cells, however each parental cell line may also show additional specific chromosome anomalies [9–12]. Taking advantage of the very strong expertise in mouse chromosome analysis available at MRC Harwell (E.P.E. and C.V.B.), we were able to identify specific chromosomal anomalies by detailed karyotype analysis of metaphase spreads [15, 19]. Table 1 summarizes the results of this comprehensive cytogenetic karyotyping screen at the MRC. These data demonstrate that four chromosomes (1, 8, 11 and Y) show markedly higher frequency of aneuploidy compared to the others. The high frequency of trisomy 8 and 11 are in-keeping with previously published studies [5, 9, 10, 12]. Over 99 % of total aneuploidy events involve at least one of these four chromosomes (1, 8, 11 and Y) in a study involving 708 clones, thus demonstrating that for JM8-derived clones, surveying these four chromosomes is sufficient to identify most clones with trisomies (Table 1). Large chromosomal fusions and translocations were also detected, but at much lower frequency compared to trisomies; 1.1 % of all clones analysed harboured translocation events, all of which involved at least one chromosome from the panel identified (Chr 1, 8, 11 and Y). The majority of translocation events detected were not balanced and/or coupled with additional trisomy events and, as a result, the copy number of the chromosomes affected will be increased and thus detectable by quantitative PCR-based methods. This indicates that precise quantification of gain or loss of these chromosomes should be sufficient to evaluate GLT potential of the great majority of clones that transit the ES cell to mouse conversion pipeline. It remains noteworthy that these methods are not expected to detect local chromosomal rearrangements that are out of the scope of this study.

**Table 1:** Cytogenetic karyotyping analysis of clones derived of the JM8 parental line

	Number of clones (percentage of 708 clones analysed)
≥50 % euploidy	569 (80.5 %)
<50 % euploidy^a^	138 (19.5 %)
ES clones with Chr 1 aneuploidy^b^	11 (1.6 %)
ES clones with Chr 8 aneuploidy^b^	119 (16.8 %)
ES clones with Chr 11 aneuploidy^b^	38 (5.4 %)
ES clones with Chr Y aneuploidy^b^	36 (5.1 %)
ES clones with at least four aneuploid chromosomes including 1, 8, 11 and Y	2 (0.3 %)
ES clones with three aneuploid chromosomes (at least two of these were among 1, 8, 11 and Y)	10 (1.4 %)
ES clones with two aneuploid chromosomes (at least one of these were among 1, 8, 11 and Y)	45 (6.4 %)
ES clones with one aneuploid chromosome among 1, 8, 11 and Y	81 (11.4 %)
ES clone with aneuploid chromosomes other than 1, 8, 11 or Y	1 (0.1 %)
ES clones with only aneuploid Chr 1 only	3 (0.4 %)
ES clones with only aneuploid Chr 8 only	64 (9.0 %)
ES clones with only aneuploid Chr 11 only	6 (0.8 %)
ES clones with only aneuploid Chr Y only	2 (0.3 %)

The table shows the percentage and number of clones normal or with given aneuploidy (1, 8, 11, and Y) and summarises the cytogenetic karyotype analysis data generated at MRC Harwell

^a^Those clones carrying multiple abnormalities are included in more than one category and as such the numbers recorded in the abnormality columns may exceed that recorded in the < 50 % euploidy category

^b^Only this aneuploidy or in combination with others aneuploidies

### Novel rapid method for identification of aneuploid ES cell clones

Pilot experiments to assess chromosome number were carried out using standard Taqman^®^ based quantitative Polymerase Chain Reaction (qPCR) copy counting and genomic DNA (gDNA) in crude extracts prepared from ES cells. In our hands, this technique was unable to evaluate with sufficient accuracy the contribution of euploid cells in cultured clones i.e. identifying clones with <50 % trisomy requires accurate differentiation between copy numbers of 2.4 (trisomy present in 40 % of cells) and 2.6 (trisomy present in 60 % of cells), without large numbers of replicates. Furthermore, this method did not accommodate the heterogeneity of such samples, requiring precise input calibration to compensate for the variability of cell growth between ES cell clones (Additional file 3: Figure S3). This is in keeping with previous published studies [20, 21].

ddPCR CNV experiments using two Taqman^®^ probe-based assays proved a more sensitive and flexible method for evaluating chromosome copy number from ES cell-derived gDNA [21]. The copy number of a single gene on each aneuploidy-prone chromosome (1, 8, 11 or Y; detected by a FAM**™**-labelled probe) was assayed relative to that of a calibration gene (present on either Chr 10 (MRC) or Chr 17 (ICS); detected by a VIC^**®**^-labelled probe), each shown to be generally maintained at a stable diploid state by cytogenetic karyotype analysis. Importantly, the assays proved robust with a wide range of concentrations of gDNA input (Additional file 4: Figure S4).

**Fig. 2:**
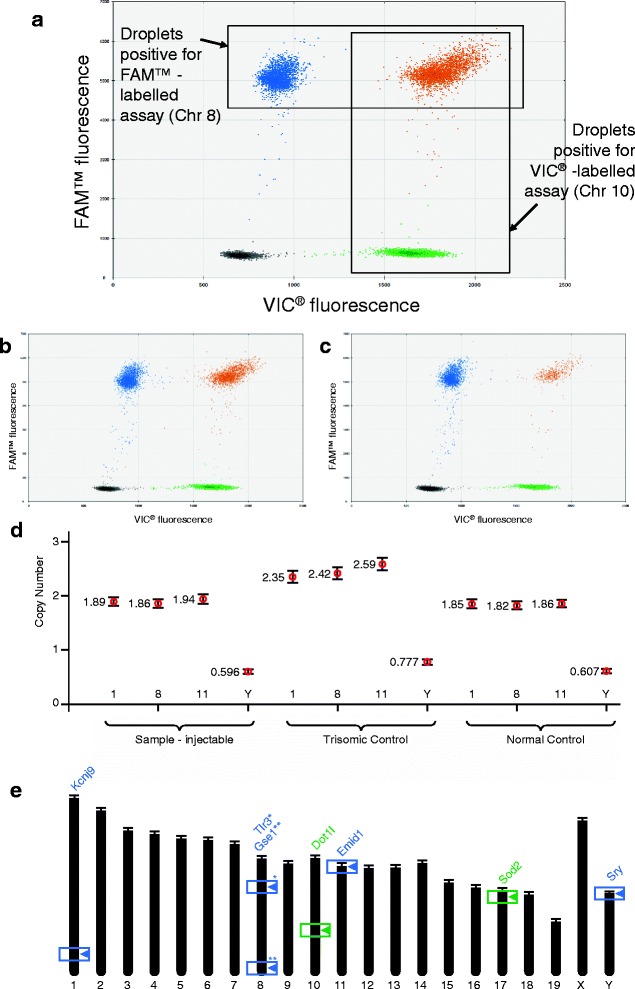
Example of evaluation of copy number by ddPCR. Panel **a** shows an annotated example of FACS-like plot obtained with the QuantaSoft software, version 1.2.10.0 (Bio-Rad, CA, USA) taken from a CNV2 copy counting ddPCR experiment. Panel **b** and **c** show typical results obtained from quantifying Chr 8 in a euploid and a trisomic sample, respectively. Panel **d** shows copy numbers as calculated and presented in the CNV option obtained with known euploid (Normal) and trisomic for Chr 1, 8 and 11 (Trisomic) samples as external calibrators. A new sample of unknown quality is shown to be injectable. Vertical bars are Standard Errors. Panel **e** presents the distribution of the marker genes and their mouse chromosomal location and the assays that were employed in this study (* and ** show the position on Chr 8 of Tlr3 and Gse1, respectively)

Figure 2a shows an example of FACS-like plot obtained when analysing ddPCR data: We designed reference assays on chromosomes that remain diploid (as identified by our karyotyping survey). Black dots represent droplets negative for both measured and reference chromosomes. Green dots represent droplets positive for the assay designed against the reference chromosome only. Blue dots represent droplets positive for the assay designed against the measured chromosome only (e.g. Chr8). Orange dots represent the droplets positive for both assays. The signal obtained from the reference chromosome is used to assess the number of genomes in the reaction. The droplets positive for the measured chromosome (Blue and Orange) in relation to those positive for the reference (Green and Orange) are employed to define the ratio between the copy numbers of these chromosomes (see Materials and Methods). This yields the copy number of the measured chromosome, as the reference chromosome is known to remain diploid and is set at 2 (CNV2 experiment). Figure 2b and c illustrate an example of ddPCR data obtained with 2 clones previously shown, by analysis of chromosome spreads, to be euploid and triploid for Chr 1, 8 and 11, respectively. Figure 2d shows respective chromosome copy numbers relative to that of a calibration gene. A range of gene assays distributed along Chr 1, 8 and 11 were tested on known samples i.e. previously analysed by cytogenetic karyotyping, before an optimal panel was chosen on the basis of reproducibility and compatibility of the optimal reaction conditions between the FAM^**™**^- and the VIC^**®**^-labelled assays and across the panel. Figure 2e illustrates the distribution of these assays on mouse chromosomes and the sequences of primers and probes are presented in Additional file 5: Table S1.

**Fig. 3:**
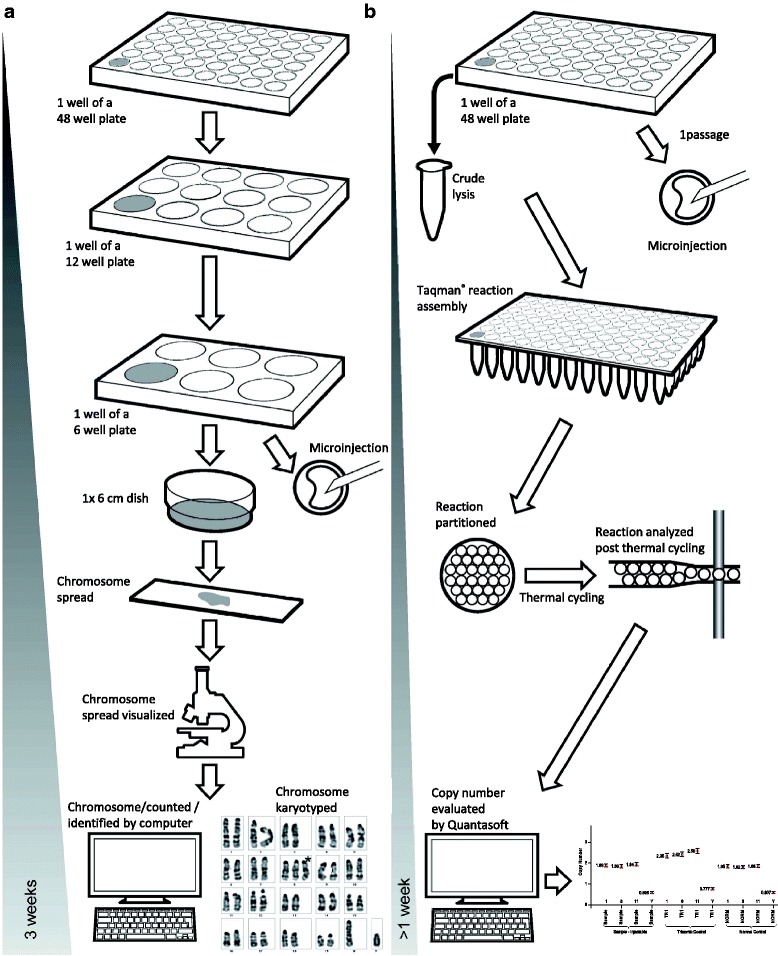
Comparison of processes based on karyotyping of mitotic chromosome spreads and ddPCR chromosome counting. Panel **a** details the method including chromosome spreads that we used for karyotyping by chromosome counting of ES cell lines. Note that the ES cell amplification phase spans several culture passages, including intensive preparation and evaluation of samples. The overall length of the process covered a period of 3 weeks for each sample. Panel **b** details the alternative process based on the novel ddPCR method introduced in this article as implemented at MRC Harwell. Note the shortened cell culture period, less intensive wet laboratory time (PCR-based), a faster readout of copy numbers from raw data with an overall process time of less than 1 week for each sample. For operational reasons, the ddPCR screen is implemented at a later passage at ICS. A key aspect of the workflow is that the DNA extraction is performed from an ES cell passage number close to that at which the cells are injected

As illustrated in Fig. 2d, we found that the ddPCR-based protocol affords the sensitivity required for the identification of clones with trisomy contribution that impede GLT capacity. Furthermore, it accommodates the variability of DNA samples obtained from clones that greatly vary in cell density and the use of a time-saving crude lysis DNA preparation method (Fig. 3), in keeping with other applications previously developed with the technique [21].

It is noteworthy that clones shown to be euploid by karyotyping of metaphase spreads typically yield ddPCR copy numbers that are lower than 2 (ranging from 1.7 to 1.9). In a similar vein, the copy numbers for the Y chromosome are routinely recorded ~0.5 and not 1, although complete loss of Y is easily identifiable by a lack of amplification of FAM^™^-labelled targets. This is not due to poorly adjusted assay efficiency, as this panel of assays run in the same conditions but using DNA extracted from ear and other biopsies typically yields a copy number of 2 (Additional file 6: Figure S5). This suggests that the slightly lower copy number found in ES cells is representative of the overall population in culture, rather than a reflection of the relative efficiencies of assays. Also, chromosome spreads count chromosomes only in the metaphase population, whereas the ddPCR surveys all the genomes present in culture. We therefore systematically include a euploid and a trisomic sample, characterised by cytogenetic karyotype analysis, in each experiment that act as calibrators. One further concern was the variability that the un-coordinated nature of DNA replication between copy-counted loci during the G2 phase could have been a confounding factor, however we found that the level of variability introduced was not sufficient to cause a discrepancy in aneuploidy calls between methodologies.

We have mixed euploid and aneuploid (Ts8, NsY) ES cells in increasing ratios and measured Chr 8 and Chr Y copy numbers (Additional file 7: Figure S6). Thus, we have illustrated that the cut-off point for microinjection (up to 50 % of euploid cells inclusive to be injected) corresponds to the midpoint between the values obtained with the control DNA that we run for each experiments (euploid and triploid clones).

Practically, several clones are often available for the same targeting event and the clone(s) with values closest to that obtained with the known euploid sample are selected for microinjection. Clones with borderline values i.e. close to cut-off threshold (light grey area), are less attractive candidates for microinjection, as they are expected to be taken over by their aneuploid sub-population as they are cultured further.

Figure 3 summarises karyotyping processes based on (a) karyotyping of metaphase chromosome spreads and (b) ddPCR and shows the marked difference of timeline between the two processes; 3 weeks and less than 1 week, respectively, with the latter requiring less amplification of materials in cell culture and less labour for implementation and analysis of assays. No chromosomal abnormalities were observed by ddPCR for 72 % of the 378 tested clones. This is in keeping with our results from karyotyping chromosome spreads that showed that ES cells clones with less than 50 % euploid metaphases represented nearly 25 % of total cells clones. For the remaining 28 % JM8-derived clones of the ddPCR study, 3, 22, 6 and 1 % of the clones show variation of Chr 1, 8, 11 and Y ratio respectively, with some clones showing more than one aneuploidy event (Data detailed in Additional file 5: Table SI). Again, this study correlates with data obtained from karyotyping of chromosome spreads where Chr 8 was identified as the major source of trisomy.

### Screening strategy

We found that assays along each chromosome could be employed interchangeably, provided that the required annealing temperatures were compatible with that of the calibration gene assay (Additional file 8: Figure S7, Additional file 5: Table S2). We validated the ddPCR method by comparing karyotyped chromosome metaphase spreads and ddPCR data obtained from 16 clones (some euploid and some aneuploid). This showed excellent correlation between the outcomes of these two methods as only one clone was differently scored by the two techniques.

Although some clones trisomic for Chr 8 also carry other chromosomal abnormalities (0.8 % clones analysed have aberrations on Chr 8 and 1, 4.1 % have trisomies on both Chr 8 and 11 and aneuploidy on Chr 8 coupled with Chr Y is detected at 4.0 %), the frequencies of such compound event suggest that these aneuploidies arise independently (See Additional file 5: Table S3). Interestingly, double trisomy of Chr 8 and 11, found at high frequencies in 2 unstable cell lines by Gaztelumendi & Nogue [10], was observed at much lower frequency in JM8 subclones which suggests that these compound rearrangements are specific to the cell lines or dependent on cell culture conditions.

These data show that for JM8 cells, determining the copy number of Chr 8 and Y represents the most accelerated screen, which should detect the majority of the aneuploidy events and enable identification of mouse colonies where female chimeras should be bred instead of males. Therefore, reducing the panel of assays to two chromosomes (i.e. 8 and Y) can be sufficient.

One hundred and thirty JM8 clones with normal karyotype by ddPCR for Chr 8 and Y were subsequently analysed by metaphase spread-based karyotyping. We found that only 3.8 % (5/130) would have been eliminated by metaphase spread-based karyotyping. In 3 out of these 5 clones, the anomaly was a chromosome translocation, which is not expected to be readily detected with a method aimed at copy counting, as it is unlikely to affect the overall copy numbers of genes assayed. Translocations without accompanying chromosome number abnormalities represent such a rare occurrence (3/130) within our large dataset, that we elected not to aim to identify them as part of our screen. Noticeably, the switch to ddPCR to identify and discard aneuploid clones from the process of ES cell to mouse conversion was not associated with any loss of GLT efficiency in the production pipeline of either centre.

However, when we applied the same approach to clones obtained from two other C57BL/6N parental lines isolated at ICS (S3 and TB1 ES, unpublished data, ICS), we found 13.5 % (10/74) and 15.6 % (7/45) of clones quality control passed by ddPCR still showed various chromosomal anomalies with metaphase spread-based karyotyping, respectively (See Additional file 5: Table S4). We therefore recommend that ddPCR-based chromosome counting should focus on detecting the range of chromosomal instabilities relevant to the parental clone and cell culture conditions employed. It also confirms that, with the exception of trisomy 8, the frequencies of all other chromosomal abnormalities are specific to the parental cell line. However, when relevant, assays developed to count chromosomes in one particular cell line would work in most other ES cell lines, as genomic sequences are greatly conserved. Also, the combination of assays presented here will be relevant to the activity of many laboratories, as it was developed to survey JM8-derived clones that constitute the two major publicly available conditional mutant collections of the field [1].

### Multiplexed ddPCR reactions for chromosome counting

**Fig. 4:**
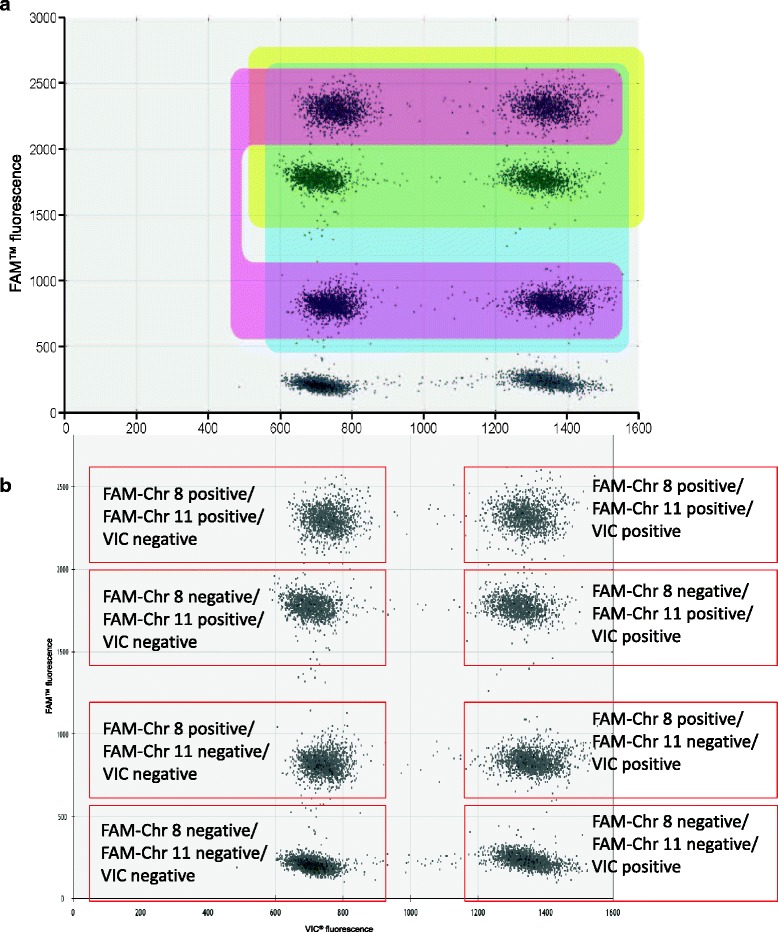
Evaluation of copy number by multiplexed ddPCR. The figure describes the structure of a FACS-like plot obtained with multiplexed ddPCR analysed with the QuantaSoft software, as in version 1.2.10.0 (Bio-Rad, CA, USA). **a** The area in blue shows the droplets positive for either or both unstable chromosomes analysed. The area highlighted in yellow shows droplets positive for the assay of Chr 11, whilst the area shaded in pink shows the droplets positive for the other unstable chromosome analysed (8); (**b**) a similar plot where each droplet populations are annotated

In order to further lower the reagent and labour costs associated with the quality control of ES cell chromosome number, we took advantage of the scope of the ddPCR format by multiplexing two Taqman® assays labelled with the same fluorophore [22]. We chose the combination of two chromosome-specific FAM^**™**^-labelled assays multiplexed with one VIC^**®**^-labelled reference assay, as the former type of probe yields a stronger signal at equivalent concentration. The outcome of such an experiment can be represented in a dotplot diagram with eight clouds, corresponding to populations of droplets in which different combinations of assays are positive (Fig. 4). Examples (including data obtained with a euploid external calibrator) are shown in Additional file 9: Figure S8. We combined assays for Chr 8 and 11 in a primary screen, thus removing most of the trisomic clones from the process in a single well. In the second round, Chr 1 and Y are assayed independently. Clones with aneuploidy of Chr 1 are discarded and females chosen for breeding among the chimeras obtained from ES cell clones lacking Chr Y if no other suitable clone is available. The assays currently used for Chr 1 and Y are not compatible with multiplexed reactions because the levels of fluorescence emitted are too similar to differentiate between assays at efficient concentrations. Alternative assays will need to be identified to allow these two chromosomes to be assayed in the same reaction. Multiplexed ddPCR assays are now integrated in our large-scale screening process, as we found that they yielded outcome comparable to those obtained with duplexed assays (See examples in Additional file 5: Table S5 and Additional file 9: Figure S8).

### Applications beyond large-scale ES cell to mouse conversion programme

The ddPCR method we present here can be widely employed. Examples include the simple quality control of wild type ES cells prior to electroporation or targeted ES cells at a point of amplification equivalent to microinjection. The protocol can also be used for the rapid screening of ES cell colonies in 96-well format, combining loss of allele assay CNV experiment following gene targeting and an assessment of aneuploidy complement. Additional potential applications cover the evaluation of copy number of endogenous genes or transgenes or karyotype variations in any other in vitro experimental models.

## Conclusions

We summarise the results of karyotype screening of over 3,500 ES cell clones, from the IMPC pipeline and additional external projects at both the MRC and ICS, by traditional or novel methodologies. We have identified the threshold of karyotypic instability affecting GLT capacity in the widely used C57BL/6N-derived cells and others as 50 % of a cell population, irrespective of the parental ES cell line involved. We showed that the majority of chromosome abnormalities found in these cells involves Chr 1, 8 and 11 trisomies and loss of Chr Y, singly or in combination. The data collected by the two centres demonstrates that ddPCR is a rapid, simple and affordable method to replace the more exacting metaphase spread-based karyotyping. Contrary to metaphase spread-based karyotyping, the ddPCR protocol is simple to set up and can be done in medium to high-throughput with basic molecular biology expertise. In our institutes, the combined labour and consumable costs per sample is approximately $30 for ddPCR karyotyping, which is tenfold lower than metaphase spread-based karyotyping ($300 per sample). Although the equipment needed to generate and analyse droplets is expensive, it is of an equivalent cost to that of a camera and analysis software used for metaphase spread-based karyotyping. We have further optimised this method, by validating ddPCR-based multiplexing of 3 assays (two targets versus one reference), with crude lysis DNA extracts from cultured cells as the template. It has proved a suitable method for the evaluation of the euploid complement of cultured cells and the identification of ES cells clones with high GLT potential and can be extended to copy number of endogenous or transgenic markers. Thus, it will reduce the number of failed attempts to obtain genetically engineered mouse models, reducing the number of animals used and so providing a clear ethical improvement compatible with the 3R’s (Reduction, Refinement, Replacement) principle [23].

## Methods

### ES cells

JM8 C57BL/6N clones were obtained from a variety of sources including both MMRRC (https://www.mmrrc.org/) and EuMMCR (https://www.eummcr.org/) repositories.

P1 129S1, S3 C57BL/6N, TB1 C57BL/6N and BD10 C57BL/6N parental lines were established at ICS and selected for low chromosomal abnormalities frequency and high GLT efficiency potential.

### Chromosome counting following Giemsa staining

ES cells grown without feeders were treated with 0.02 μg/ml colcemide for 2 h. Cells were then trypsinised, and the cell pellet was incubated in 0.56 % KCl for 20 min in 5 % CO_2_ at 37 °C (hypotonic shock). Cells were then fixed in methanol-acetic acid 3V/1V for 20 min at room temperature, then washed three times with methanol-acetic acid and concentrated in a small volume. Drops of cell suspension were then plated on glass slides at 50 °C. The cells were then allowed to dry and stained with Giemsa 4 %. 30 metaphases are analysed using Ikaros software (MetaSystems Hard & Software, Germany). Examples of data obtained are shown in Additional file 10: Figure S9 panels A and B.

### Karyotype analysis of ES cells

Cells grown without feeders were treated with 0.02 μg/ml colcemide for 2 h and then treated as in [19] to obtain mitotic chromosome spreads on glass microscope slides for karyotyping. For identification of chromosomes, either of the following methods was used: (a) Images of DAPI stained mitotic chromosome spreads were captured using a fluorescence microscope with Smartcapture 2 software (Digital Scientific, Cambridge, UK). The DAPI staining on the chromosomes was reversed, and enhanced by the software to produce reversed DAPI banding (or pseudo G-bands) that are equivalent to Giemsa stained G-bands. This allowed comparison with the standard ideogram of the mouse [15] to precisely determine the origin of any aneuploidy or other chromosome anomalies identified in each mitotic spread. Examples of data obtained are shown in Additional file 10: Figure S9, panels C and D; or (b) G-banded [19] chromosome mitotic spreads from approximately half of the ES cell clones were analysed using a standard laboratory microscope for aneuploidy or other chromosome anomalies. Initially 50 metaphase spreads were analysed from each ES cell line. This was later reduced to 30 cells per cell line with no change in the accuracy of results.

### Microinjection and germline transmission

Targeted ES cell clones obtained from the EUCOMM and KOMP cell repositories (EuMMCR and MMRRC) or produced in house by either MRC or ICS, were injected into BALB/cAnN or C57BL/6J blastocysts for chimera generation. The resulting chimeras were mated to C57BL/6N mice, and the progeny were screened to confirm GLT.

### Genomic DNA lysate preparation

We prepared crude lysis extracts from cultured cells (minimum of a 1/10th a semi-confluent well of a 6-well plate, corresponding to less than 1 well of a 48-well plate). Cells were scraped from the plate surface in PBS and pelleted by centrifugation and the PBS removed. The cell pellets were lysed in at least 30 μl of lysis buffer (Taqman^®^ Sample-to-SNP kit, Applied Biosystems) and processed as per manufacturer’s instructions. Extraction was performed at 95 °C in a thermocycler for 3 min prior to addition of 30 μl stabilizing buffer. Samples were stored at 4 °C for up to two weeks, and frozen if long term storage was required. Alternatively (at ICS), following digestion with proteinase K, the genomic DNA was extracted with phenol/chloroform followed by ethanol-precipitation, as described [24].

### Taqman^®^ assays

We obtained qPCR assays from Biosearch Technologies, CA or synthesized as PrimeTime Assays by Integrated DNA Technologies. Hydrolysis probes contained a 5’FAM^**™**^ fluorophore with either an internal ZEN and a 3-Iowa black quencher or a 3’ BHQ1 quencher. Sequences of primers and probes are listed in Additional file 5: Table S1.

### Droplet digital PCR reactions

For duplex reactions, when a single chromosome target was amplified in parallel with a reference gene assay, a copy number variation experiment with the reference set at 2 copies (euploid, CNV2) on the Bio-Rad QX200 ddPCR system (Bio-Rad, CA) was performed. Reaction mixtures (20 μl) contained 1 μl crude DNA lysate or 50 ng of phenol/chloroform purified genomic DNA, 1x ddPCR Supermix for probes (Bio-Rad, CA, USA), 225 nM of each primer (two primers per assay used) and 50 nM of each probe (one VIC^**®**^-labelled probe for the reference gene assay and one FAM^**™**^-labelled for the chromosome target assay). These reaction mixes were loaded either into DG8 cartridges together with 70 μl droplet oil per sample and droplets generated using the QX100 Droplet Generator or loaded in plate format into the Bio-Rad QX200 AutoDG and droplets generated as per the manufacturer’s instructions. Post droplet generation, the oil/reagent emulsion was transferred to a 96 well semi-skirted plate (Eppendorf AG, Hamburg, Germany) and the samples were amplified on the Bio-Rad C1000 Touch thermocycler (95 °C for 10 min, followed by 40 cycles of 94 °C for 30 s and 58 °C for 60 s, with a final step of 98 °C for 10 min). The plate containing the droplet amplicons was subsequently loaded into the QX200 Droplet Reader (Bio-Rad, CA, USA). Standard reagents and consumables supplied by Bio-Rad were used, including cartridges and gaskets, droplet generation oil and droplet reader oil. For multiplex reactions, the recipe was altered to reflect the presence of another primer pair and FAM^**™**^-labelled probe. Reaction (20 μl in total) contained 1 μl crude DNA prep, 1x ddPCR Supermix for probes (Bio-Rad, CA, USA), 112.5 nM of each chromosome-specific primer (a primer pair for Chr 8 and 11), 25 nM of each FAM^**™**^-labelled probe, 250 nM of each reference gene primer and 50 nM of VIC^**®**^-labelled reference gene probe. Droplets were generated as previously described and subject to the same thermal cycling conditions before being analysed on the QX200 Droplet reader.

### Analysis of ddPCR data

The QX200 Droplet Reader recorded the number of accepted droplets in which fluorescence within the droplet is detected (positive droplets) by either one or both optical channels, whilst also counting the number of droplets in which fluorescence is absent (negative droplets). A minimum of 10,000 accepted droplets per sample were used for analysis. Optimised amplification of each of the two assays produced a 2D plot of amplitude showing four distinct groups of data points corresponding to FAM^**™**^ negative/VIC^**®**^ negative, FAM^**™**^ negative/VIC^**®**^ positive, FAM^**™**^ positive/VIC^**®**^ negative and FAM^**™**^ positive/VIC^**®**^ positive droplets (Fig. 2a). The number of droplets recording fluorescence for the chromosome-specific assay was compared to the count obtained for the reference-specific assay, known to occur as two copies per genome. Final copy numbers were calculated employing the manufacturer’s QuantaSoft Software (Bio-Rad, CA, USA) by applying Poisson statistics to the fraction of end-point positive reactions (18). For further confirmation of copy number calls, we also ran a known trisomic and known normal control.

For multiplex reactions, the 2D plot now displays eight distinct groups of data points, which correspond to the different combinations of each assays being positive or negative (d escribed in details in Fig. 4). As the fluorescence profile i.e. level of amplitude recorded is largely unchanged for each assay when multiplexed, it is possible to determine which groups are associated with each assay and as such it is possible to assign results to each assay individually compared to the reference, whilst running in multiplex. To assign droplets to assays, the cross-hair or lasso tool was employed to select specific groups of data points. However, as the software only allows the definition of four categories of data points, assays must be analysed iteratively. Overall copy numbers of both the chromosome-specific assays compared to the reference are calculated based on the six higher groups of the plot combined (blue area in Fig. 4), while the copy number of Chr 11 is calculated based on the four higher groups of the plot combined (yellow area in Fig. 4). The copy number of the Chr 8 is calculated by the difference of total and Chr 11 numbers (pink area in Fig. 4). Once again, external calibrators, i.e. wells containing a known trisomic and normal control samples were included (see examples in Additional file 9: Figure S8). Data was exported from the Quantasoft software following each round of analysis to give complete profile for each sample.

## Abbreviations

Chr, chromosome; CNV, copy number variation; ddPCR, droplet digital polymerase chain reaction; DNA, Deoxyribonucleic Acid; ES, Embryonic Stem; G-banding, Giemsa banding; gDNA, genomic DNA; GLT, Germline Transmission; ICS, Institut Clinique de la Souris; IMPC, International Mouse Phenotyping Consortium; MLC, Mary Lyon Centre; MRC, Medical Research Council; qPCR, quantitative Polymerase Chain Reaction

## Additional files

Additional file 1: Figure S1. Analysis of clones of a variety of genetic backgrounds by chromosome counting in metaphase chromosome spreads. Percentage of euploid metaphases observed by Giemsa staining metaphase spread-based karyotyping was compared to germ line efficiency obtained at ICS. Data for 3 different ES cells lines, (197 XG clones, 386 BD10 clones and 284 P1 clones) from the C57BL/6N or 129S1 background are presented. Analysis using the Fisher Exact test yielded P values of 0.016083, 0.132600 and 0.117647 for XG, BD10 and P1, respectively. False discovery rate calculated by the Benjamini-Hochberg procedure (Q) were 0.03216, 0.1326 and 01326, respectively. This showed that clones with greater than 50% euploid representation are preferable candidates for microinjection in the case of XG clones. The two other cell lines tended towards the same conclusion, although the low number of clones with poorer karyotype injected did not allow reaching statistical significance. As all other data obtained in this study (and others) yielded similar conclusions, we estimated that injecting more clones of poor karyotype to reach higher statistical significance for these additional cell lines would be an unethical use of animals. (PDF 123 kb) Additional file 2: Figure S2. Quality of karyotype of JM8-derived clones is equivalent among distributing repositories. The figure shows the percentages of euploid clones sourced from the two main distributors of JM8-derived cells and GLT rate obtained with them. The numbers of clones in each instance is shown. These numbers illustrate that materials obtained from different distributors are of similar quality in terms of karyotype and GLT ability. Data was analysed using the Fisher Exact test that and showed no evidence of difference of quality between the two distributors. (PDF 77 kb) Additional file 3: Figure S3. Use of standard real time PCR for chromosome counting. The figure shows copy number of Chr 8 measured by qPCR with various input quantities of genomic DNA as template. The experiment demonstrates that although there is linearity across the range of concentrations relevant to the DNA preparations assayed, the assay is not sufficiently robust for the screen. Error bar amplitude varies with gDNA input and standardizing input is challenging due to the disparity of growth rates between ES cell clones. qPCR assays were performed in triplicates. SEM are represented by error bars. Both literature and our own experience concur in concluding that standard qPCR does not allow for sufficient accuracy to reliably identify clones worthy of microinjection. (PDF 25 kb) Additional file 4: Figure S4. DNA input in ddPCR reaction. The figure shows copy number of Chr 8 (crosses) and Y (squares) measured by ddPCR with various input quantities of genomic DNA template. Vertical bars are Standard Errors. The experiment demonstrates linearity across the range of concentrations relevant to the DNA preparations assayed. This is a key point for the robustness of the screen, as gDNA preparations are challenging to standardize due to the disparity of growth rates between ES cell clones. (PDF 26 kb) Additional file 5:
**Table S1.** Sequences of primers and probes employed in this study. **Table S2.** Sequences of primers and probes evaluated for setting up screening panel. **Table S3.** ddPCR chromosome counting analysis of ES cells derived of the JM8 parental line. **Table S4.** Karyotypic anomalies in non-JM8 derived clones. **Table S5.** Comparison of ddPCR chromosome counting outcome when performed in duplex or multiplex. (DOCX 48 kb) Additional file 6: Figure S5. Outcome of chromosome counting by ddPCR with genomic DNA extracted from ear biopsies. The figure shows copy numbers obtained using the karyotype screen assay panel on DNA extracted from euploid ES cells (C), trisomic ES cells (Ts), X0 ES cells (Ns Y) and female mouse ear clip (E), using the same lysis method. Vertical bars are Standard Errors. The data illustrate that the assays are able detect the expected copy numbers on gDNA extracted from tisssues (2 Chr 1, 8 and 11) and that the number of these chromosomes is lower in genomes extracted from tissue cultures. (PDF 63 kb) Additional file 7: Figure S6. Cut-off value of ddPCR for detection of aneuploidy ES cell clones. We have mixed euploid and aneuploid (Ts8, NsY) ES cells in increasing ratios. The figure shows the ddPCR measurements for Chr 8 (squares) and Chr Y (circles). The horizontal axis shows the percentage of aneuploid lysate in the mix. The data illustrate that the midpoint between the values obtained with 100% euploid and aneuploid samples corresponds to the cut-off between population that are or are not worth microinjecting. (PDF 30 kb) Additional file 8: Figure S7. Outcome of copy counting by ddPCR with assays from different locations on a chromosome. Panel A shows the positions of Chr 8 assays within the chromosome (Primpol) and distally (Gse1). Panel B illustrates that different assays yield lesser (Primpol) or better (Gse1) efficiency of resolution between negative and positive droplets with cycling conditions optimized for the internal calibrator assay (Dot1l). Panel C shows comparable outcome with either assays: trisomic sample (Ts8) shows high copy number while external euploid control (C) shows copy number slightly below 2 (Vertical bars are Standard Errors.). We therefore elected to employ the latter assay for routine screening because of their compatibility for multiplexing (common optimal annealing temperature and good dot cloud resolution). (PDF 101 kb) Additional file 9: Figure S8. Multiplex assays of a euploid and a trisomic ES cell clone. Chr 8 and Chr 11 were assayed each in duplex with a reference gene assay as internal calibrator (Dot1l) (A, B, G, H) or together in multiplex with the internal calibrator (Dot1l) (C-E and I-K) in an euploid (A-F) and a Ts8 (G-L). Vertical bars in F and L are Standard Errors. Panels D, E, J and K show assays run in multiplex but where channels are selected so only the positive dots corresponding to one of the 2 target chromosome are counted. Blue and Orange are positive for the considered target chromosome(s) while Black and Green are negative. Orange and Green are positive for the Dot1l assay (internal calibrator), while Blue and Black are negative. F and L show the copy numbers resulting from these analyses, allowing the comparison of assays run in duplex and in multiplex. When all channels are tuned so all positive droplets are counted, the resulting copy number is the sum of the copy numbers of Chr 8 and Chr 11. (PDF 681 kb) Additional file 10: Figure S9. Examples of cytogenetic analysis data. Panels A and B show micrographs obtained after Giemsa staining for evaluation of euploidy by chromosome counting. Panels C and D show micrographs obtained after DAPI staining for identification of chromosomes by their banding pattern. Normal (A and C) and aneuploid (Dup1, Ts8; B and D) clones are presented. Red arrowheads point to tandem translocation resulting from chromosomal duplication. (PDF 96 kb)
