# Supplementary figures and images for: Aneuploidy screening of embryonic stem cell clones by metaphase karyotyping and droplet digital polymerase chain reaction
Source: BMC Cell Biol. 2016 Aug 5;17:30. doi: 10.1186/s12860-016-0108-6 (PMC4974727; doi:10.1186/s12860-016-0108-6)

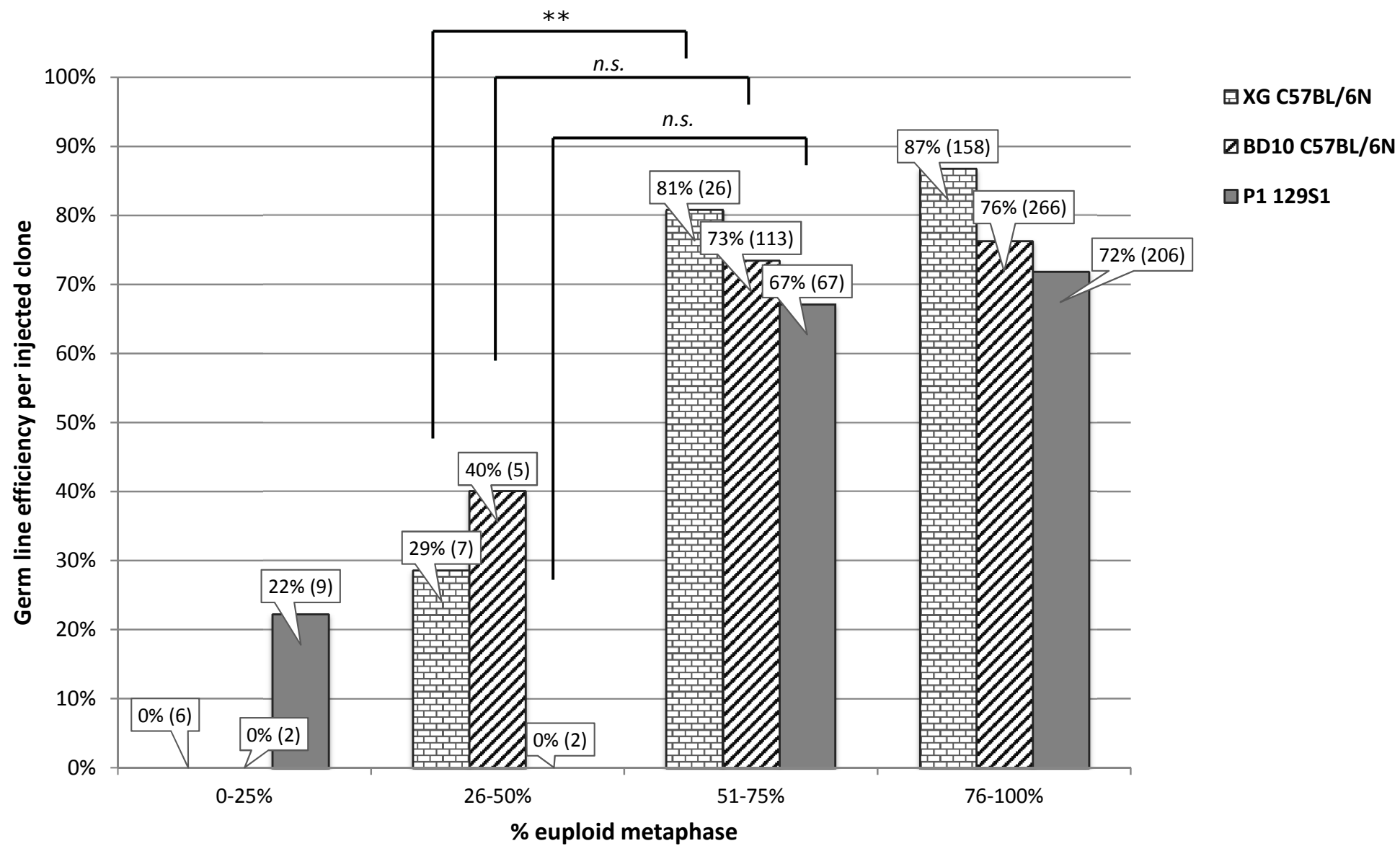

Supplement: Additional file 1: Figure S1. — Analysis of clones of a variety of genetic backgrounds by chromosome counting in metaphase chromosome spreads. Percentage of euploid metaphases observed by Giemsa staining metaphase spread-based karyotyping was compared to germ line efficiency obtained at ICS. Data for 3 different ES cells lines, (197 XG clones, 386 BD10 clones and 284 P1 clones) from the C57BL/6N or 129S1 background are presented. Analysis using the Fisher Exact test yielded P values of 0.016083, 0.132600 and 0.117647 for XG, BD10 and P1, respectively. False discovery rate calculated by the Benjamini-Hochberg procedure (Q) were 0.03216, 0.1326 and 01326, respectively. This showed that clones with greater than 50% euploid representation are preferable candidates for microinjection in the case of XG clones. The two other cell lines tended towards the same conclusion, although the low number of clones with poorer karyotype injected did not allow reaching statistical significance. As all other data obtained in this study (and others) yielded similar conclusions, we estimated that injecting more clones of poor karyotype to reach higher statistical significance for these additional cell lines would be an unethical use of animals. (PDF 123 kb) [file 12860_2016_108_MOESM1_ESM.pdf]

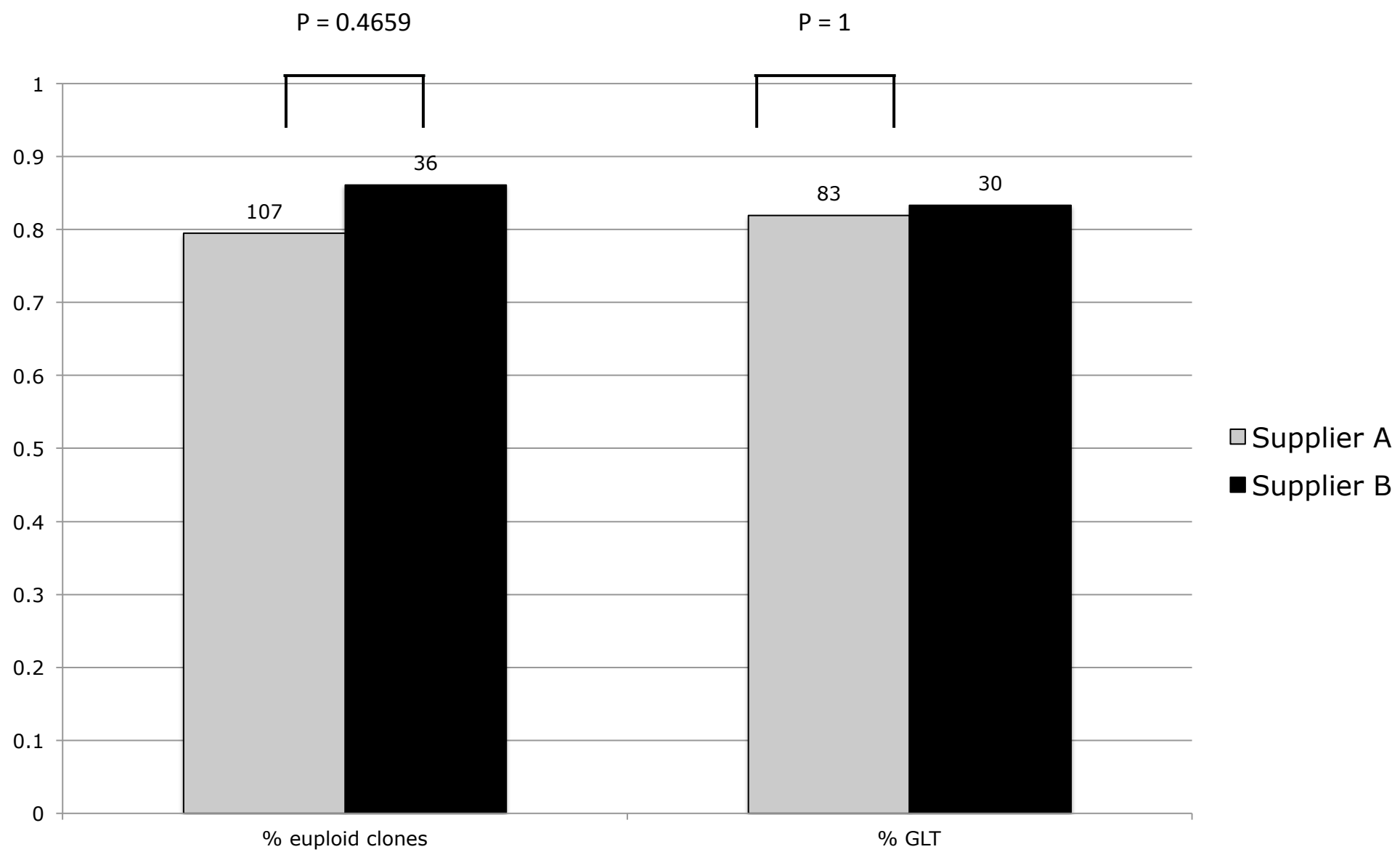

Supplement: Additional file 2: Figure S2. — Quality of karyotype of JM8-derived clones is equivalent among distributing repositories. The figure shows the percentages of euploid clones sourced from the two main distributors of JM8-derived cells and GLT rate obtained with them. The numbers of clones in each instance is shown. These numbers illustrate that materials obtained from different distributors are of similar quality in terms of karyotype and GLT ability. Data was analysed using the Fisher Exact test that and showed no evidence of difference of quality between the two distributors. (PDF 77 kb) [file 12860_2016_108_MOESM2_ESM.pdf]

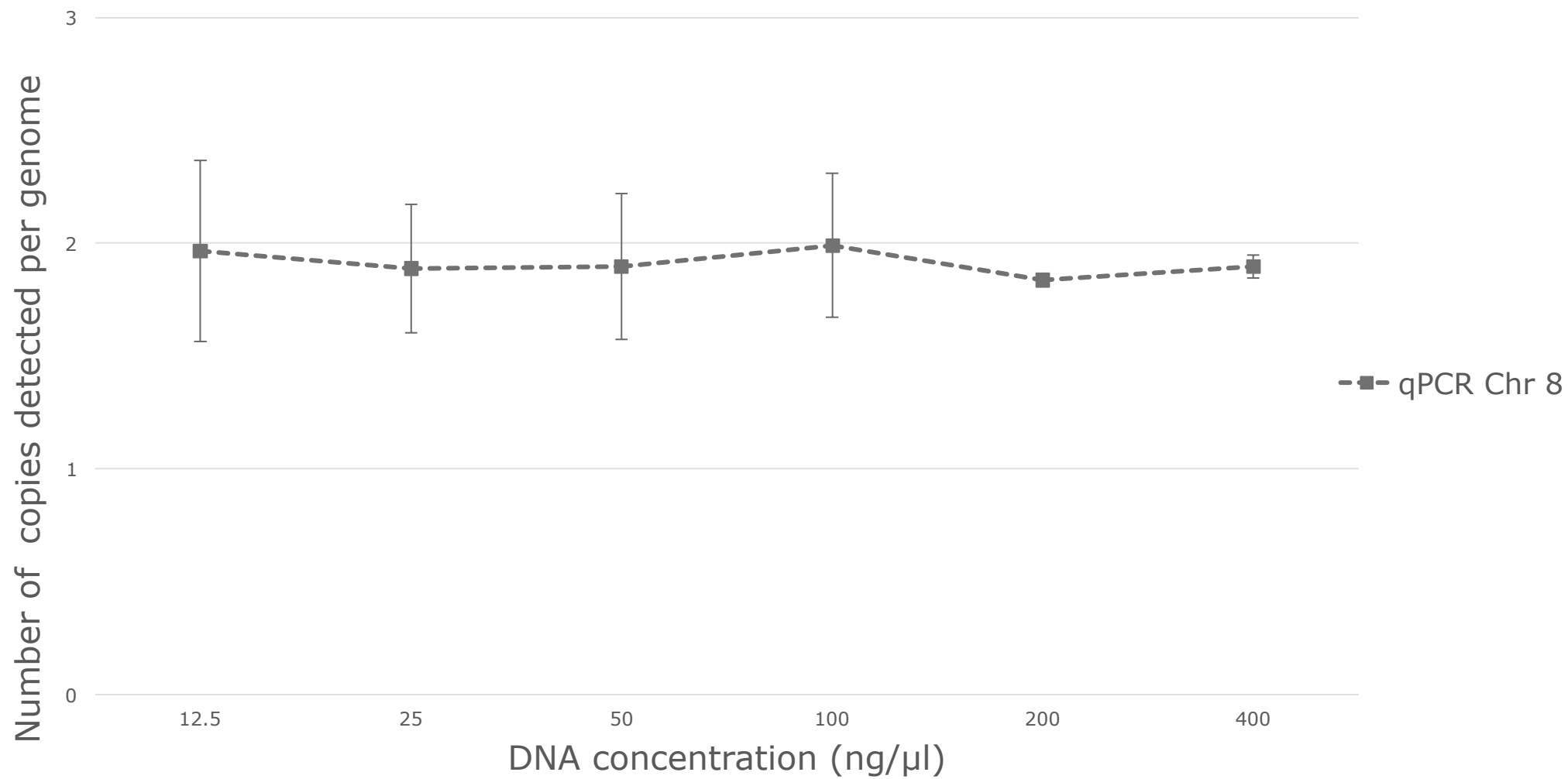

Supplement: Additional file 3: Figure S3. — Use of standard real time PCR for chromosome counting. The figure shows copy number of Chr 8 measured by qPCR with various input quantities of genomic DNA as template. The experiment demonstrates that although there is linearity across the range of concentrations relevant to the DNA preparations assayed, the assay is not sufficiently robust for the screen. Error bar amplitude varies with gDNA input and standardizing input is challenging due to the disparity of growth rates between ES cell clones. qPCR assays were performed in triplicates. SEM are represented by error bars. Both literature and our own experience concur in concluding that standard qPCR does not allow for sufficient accuracy to reliably identify clones worthy of microinjection. (PDF 25 kb) [file 12860_2016_108_MOESM3_ESM.pdf]

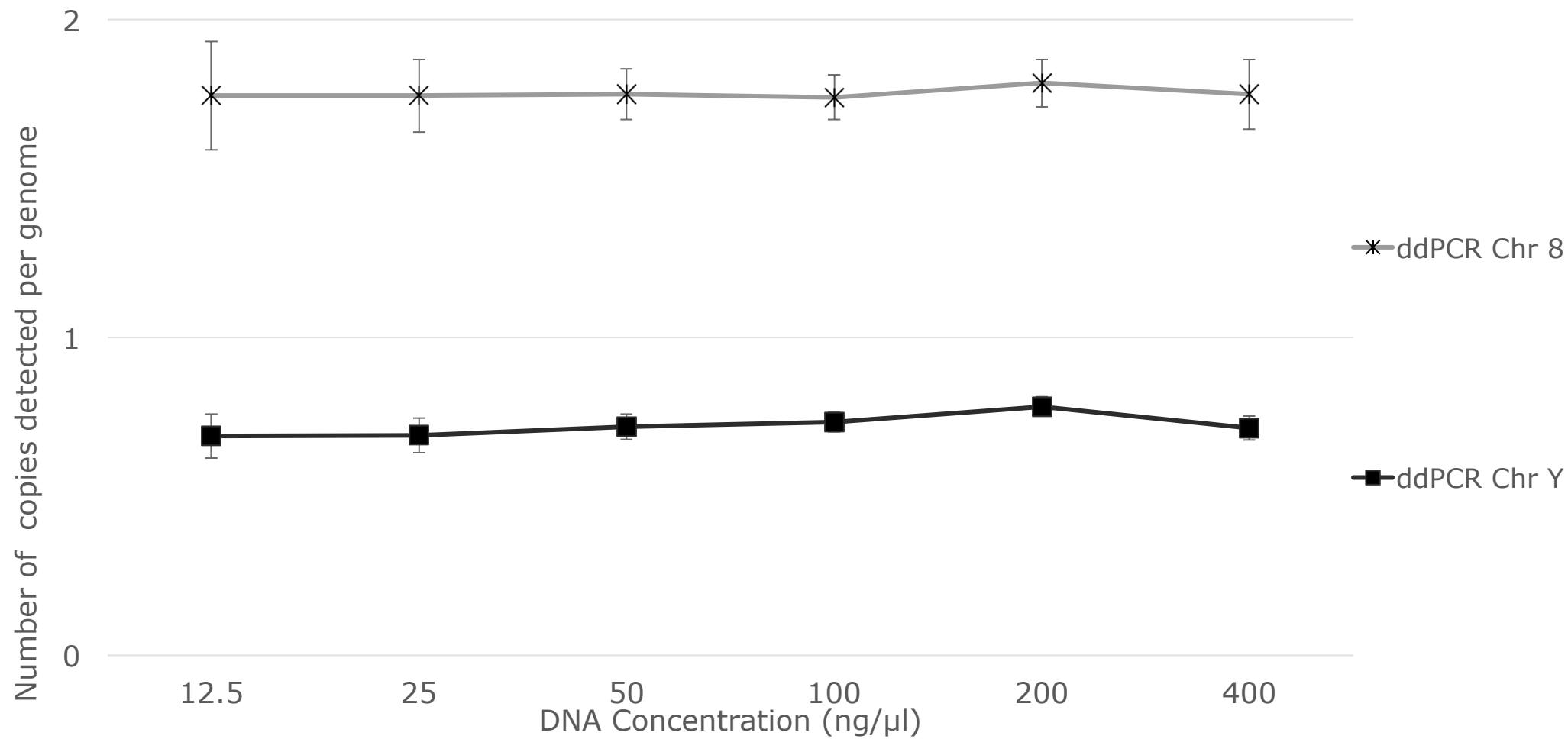

Supplement: Additional file 4: Figure S4. — DNA input in ddPCR reaction. The figure shows copy number of Chr 8 (crosses) and Y (squares) measured by ddPCR with various input quantities of genomic DNA template. Vertical bars are Standard Errors. The experiment demonstrates linearity across the range of concentrations relevant to the DNA preparations assayed. This is a key point for the robustness of the screen, as gDNA preparations are challenging to standardize due to the disparity of growth rates between ES cell clones. (PDF 26 kb) [file 12860_2016_108_MOESM4_ESM.pdf]

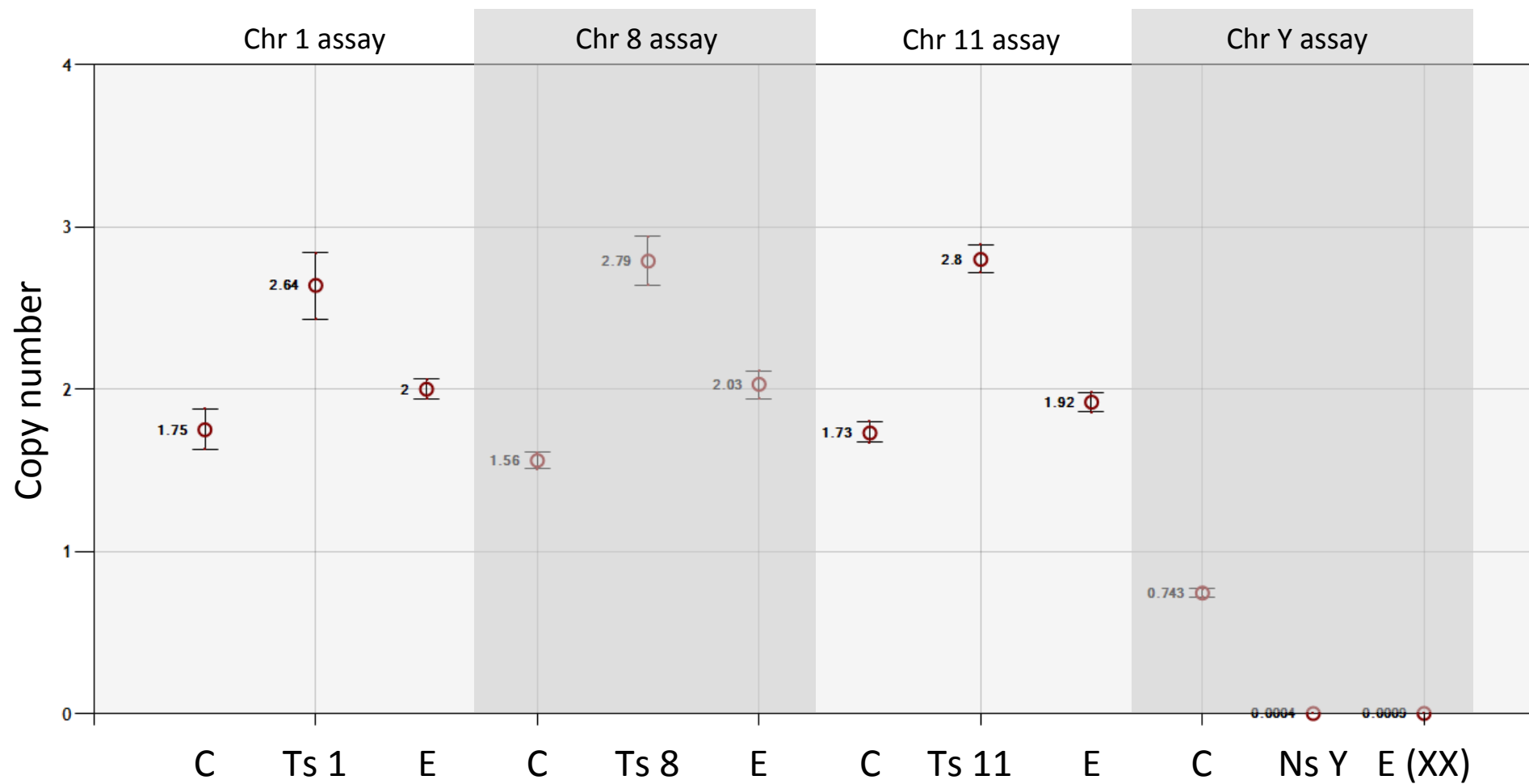

Supplement: Additional file 6: Figure S5. — Outcome of chromosome counting by ddPCR with genomic DNA extracted from ear biopsies. The figure shows copy numbers obtained using the karyotype screen assay panel on DNA extracted from euploid ES cells (C), trisomic ES cells (Ts), X0 ES cells (Ns Y) and female mouse ear clip (E), using the same lysis method. Vertical bars are Standard Errors. The data illustrate that the assays are able detect the expected copy numbers on gDNA extracted from tisssues (2 Chr 1, 8 and 11) and that the number of these chromosomes is lower in genomes extracted from tissue cultures. (PDF 63 kb) [file 12860_2016_108_MOESM6_ESM.pdf]

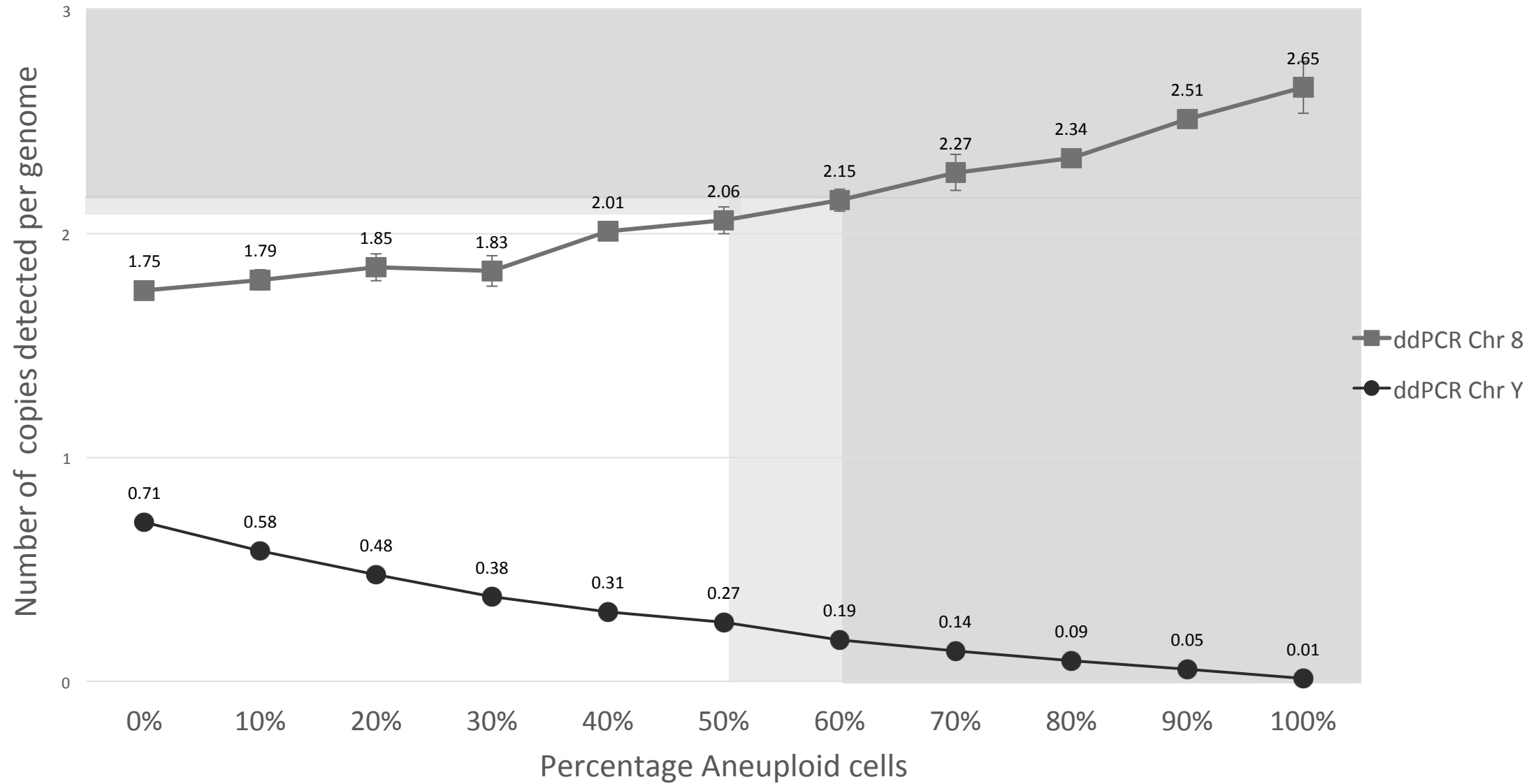

Supplement: Additional file 7: Figure S6. — Cut-off value of ddPCR for detection of aneuploidy ES cell clones. We have mixed euploid and aneuploid (Ts8, NsY) ES cells in increasing ratios. The figure shows the ddPCR measurements for Chr 8 (squares) and Chr Y (circles). The horizontal axis shows the percentage of aneuploid lysate in the mix. The data illustrate that the midpoint between the values obtained with 100% euploid and aneuploid samples corresponds to the cut-off between population that are or are not worth microinjecting. (PDF 30 kb) [file 12860_2016_108_MOESM7_ESM.pdf]

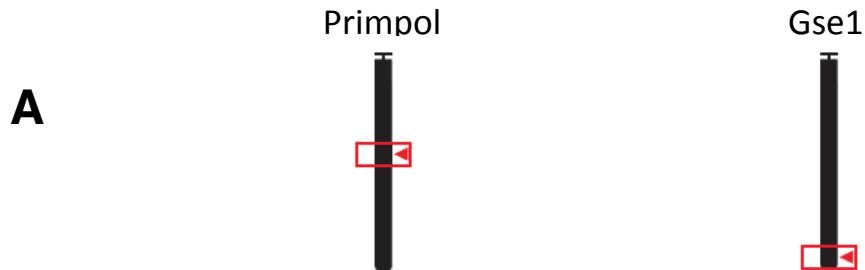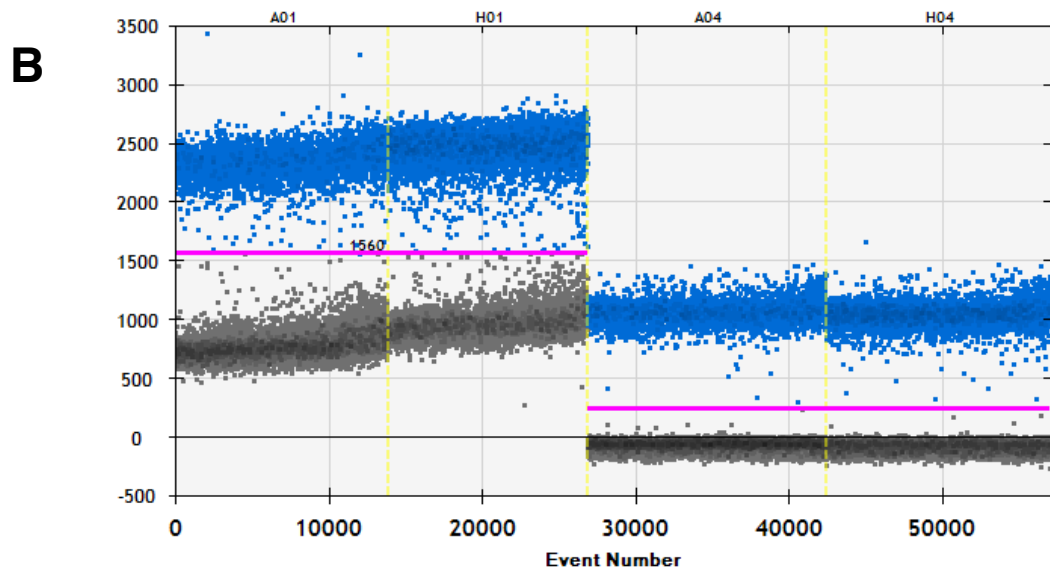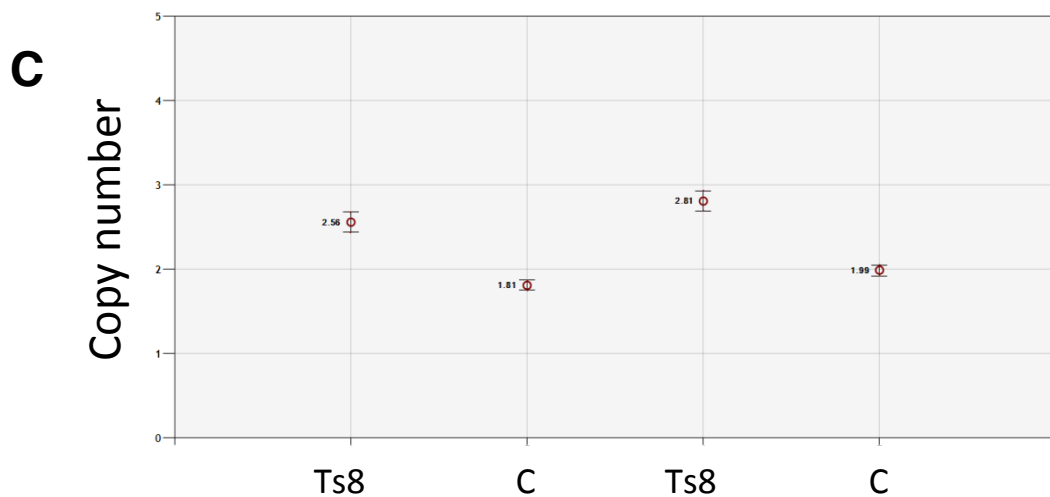

Supplement: Additional file 8: Figure S7. — Outcome of copy counting by ddPCR with assays from different locations on a chromosome. Panel A shows the positions of Chr 8 assays within the chromosome (Primpol) and distally (Gse1). Panel B illustrates that different assays yield lesser (Primpol) or better (Gse1) efficiency of resolution between negative and positive droplets with cycling conditions optimized for the internal calibrator assay (Dot1l). Panel C shows comparable outcome with either assays: trisomic sample (Ts8) shows high copy number while external euploid control (C) shows copy number slightly below 2 (Vertical bars are Standard Errors.). We therefore elected to employ the latter assay for routine screening because of their compatibility for multiplexing (common optimal annealing temperature and good dot cloud resolution). (PDF 101 kb) [file 12860_2016_108_MOESM8_ESM.pdf]

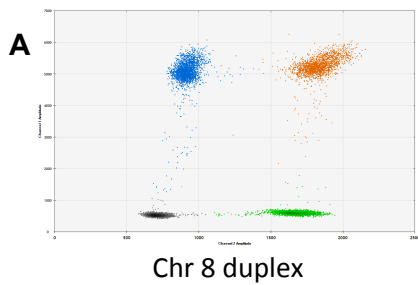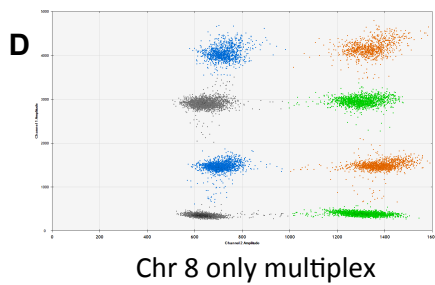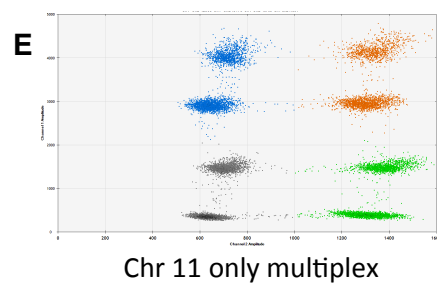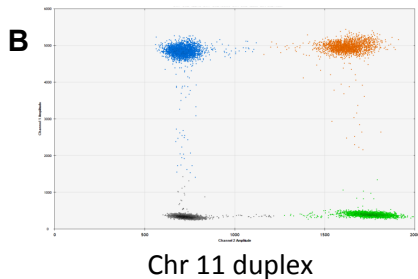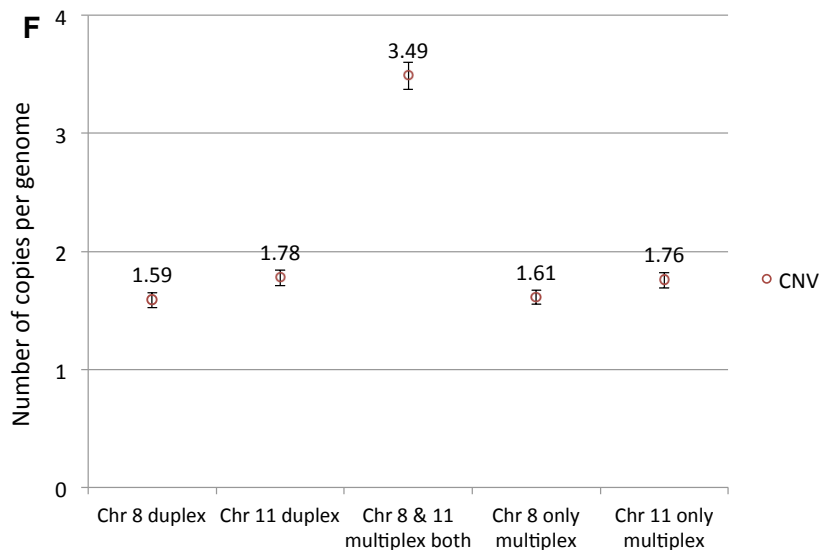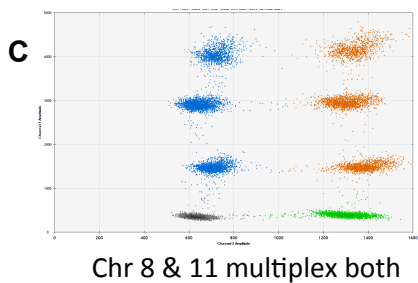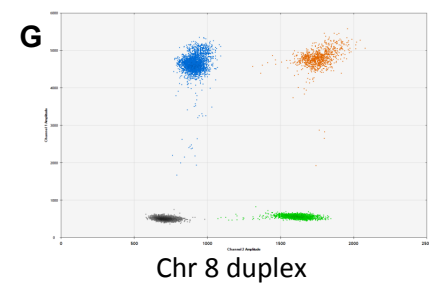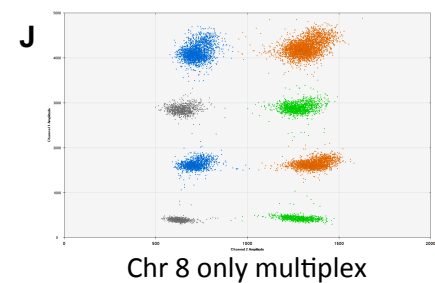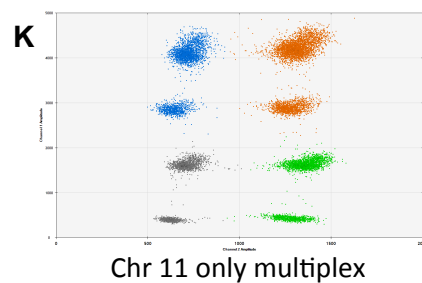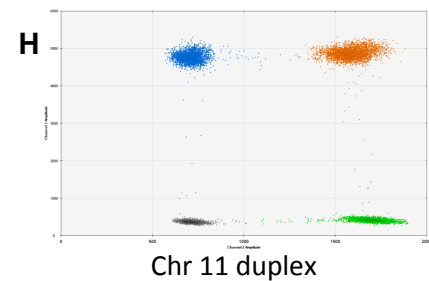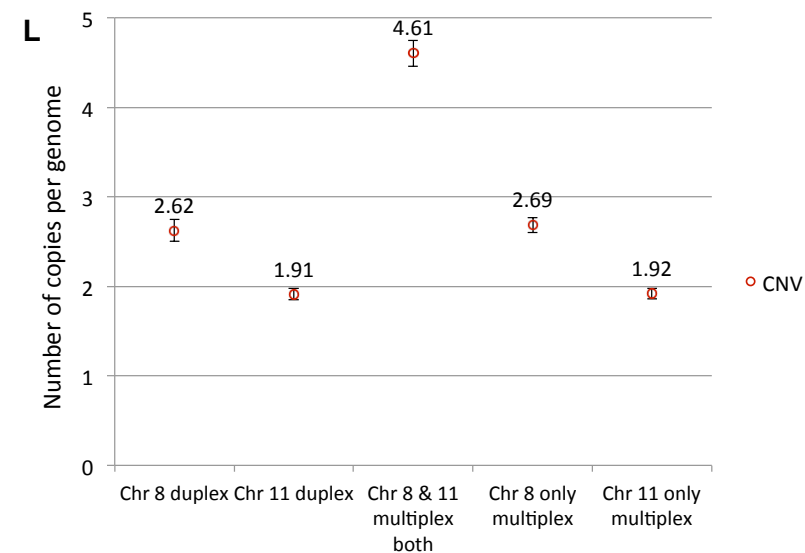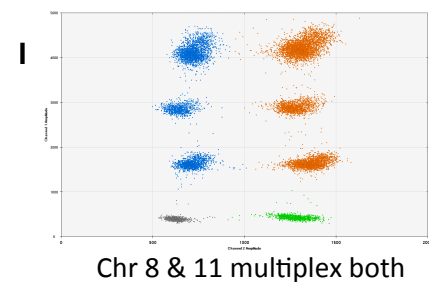

Supplement: Additional file 9: Figure S8. — Multiplex assays of a euploid and a trisomic ES cell clone. Chr 8 and Chr 11 were assayed each in duplex with a reference gene assay as internal calibrator (Dot1l) (A, B, G, H) or together in multiplex with the internal calibrator (Dot1l) (C-E and I-K) in an euploid (A-F) and a Ts8 (G-L). Vertical bars in F and L are Standard Errors. Panels D, E, J and K show assays run in multiplex but where channels are selected so only the positive dots corresponding to one of the 2 target chromosome are counted. Blue and Orange are positive for the considered target chromosome(s) while Black and Green are negative. Orange and Green are positive for the Dot1l assay (internal calibrator), while Blue and Black are negative. F and L show the copy numbers resulting from these analyses, allowing the comparison of assays run in duplex and in multiplex. When all channels are tuned so all positive droplets are counted, the resulting copy number is the sum of the copy numbers of Chr 8 and Chr 11. (PDF 681 kb) [file 12860_2016_108_MOESM9_ESM.pdf]

A

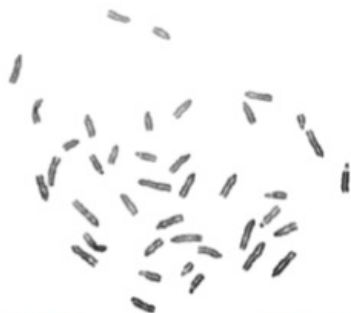

B

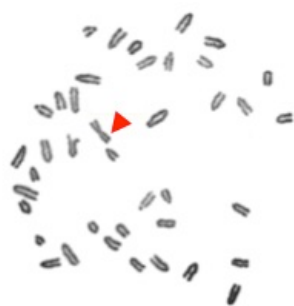

C

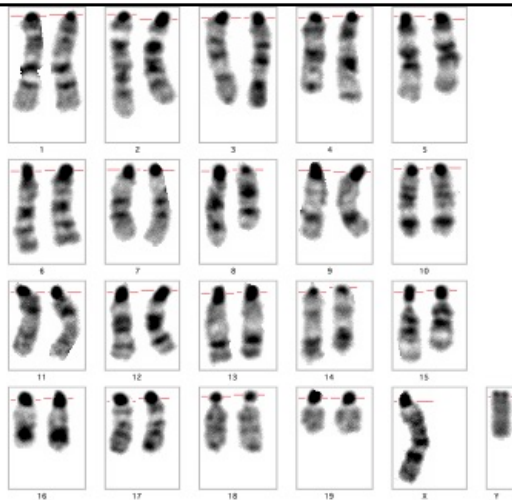

D

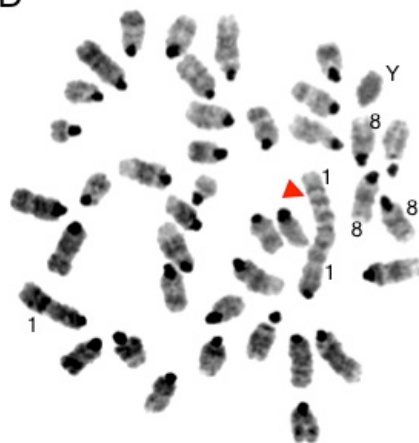

Supplement: Additional file 10: Figure S9. — Examples of cytogenetic analysis data. Panels A and B show micrographs obtained after Giemsa staining for evaluation of euploidy by chromosome counting. Panels C and D show micrographs obtained after DAPI staining for identification of chromosomes by their banding pattern. Normal (A and C) and aneuploid (Dup1, Ts8; B and D) clones are presented. Red arrowheads point to tandem translocation resulting from chromosomal duplication. (PDF 96 kb) [file 12860_2016_108_MOESM10_ESM.pdf]
