# Supplementary material for: Aneuploidy screening of embryonic stem cell clones by metaphase karyotyping and droplet digital polymerase chain reaction
Source: BMC Cell Biol. 2016 Aug 5;17:30. doi: 10.1186/s12860-016-0108-6 (PMC4974727; doi:10.1186/s12860-016-0108-6)
Supplement: Additional file 5: — Table S1. Sequences of primers and probes employed in this study. Table S2. Sequences of primers and probes evaluated for setting up screening panel. Table S3. ddPCR chromosome counting analysis of ES cells derived of the JM8 parental line. Table S4. Karyotypic anomalies in non-JM8 derived clones. Table S5. Comparison of ddPCR chromosome counting outcome when performed in duplex or multiplex. (DOCX 48 kb) [file 12860_2016_108_MOESM5_ESM.docx]

**Supplemental Table ST1: Sequences of primers and probes employed in this study.**

This table gathers the sequence details of all Taqman assays used in this ddPCR study by both centres. Probe types and assay locations differ between centres for the references used and for Chromosome 8.

| Chromosome | Centre | Gene |  | Sequence (5’-3) |
| --- | --- | --- | --- | --- |
| 1 | MRC and ICS | Kcnj9 | Primer F | CCCCTGGTGTTGCCATTTCTAG |
|  |  |  | Primer R | GACGGCTAGGACCTCTTCA |
|  |  |  | *Probe* | 5’ FAM**™**/TCACTCAGGCCTACACTGTCAAGA/BHQ-1 |
| 8 | MRC | Gse1 | Primer F | ATACGCACCCGGTACTGGAA |
|  |  |  | Primer R | GCAAGCAACGATGAATTTGTGG |
|  |  |  | *Probe* | 5’ FAM**™**/AGGATTAAGGCAACCCTTACATCAGAC/BHQ-1 |
| 8 | ICS | Tlr3 | Primer F | GACGCACCTGTTCTCTATCTG |
|  |  |  | Primer R | TTTTCCCCTTCTCCCAATACC |
|  |  |  | *Probe* | 5’6- FAM**™**/CCTGAATCA/Zen/CAATCGCGCACCAAA/3IABkFQ/ |
| 10 (reference) | MRC | Dot1l | Primer F | GCCCCAGCACGACCATT |
|  |  |  | Primer R | TAGTTGGCATCCTTATGCTTCATC |
|  |  |  | *Probe* | 5’VIC**^®^**/CCAGCTCTCAAGTCG/MGB |
| 11 | MRC and ICS | Emid1 | Primer F | GCCAGGACTGGGTAGCAC |
|  |  |  | Primer R | AGGAGGCTCCTGAATTTGTGACAAG |
|  |  |  | *Probe* | 5’ FAM**™**/CCTGGGTCATCTGAGCTGAGTCC/BHQ-1 |
| 17 (reference) | ICS | Sod2 | Primer F | AAGGAGCAAGGTCGCTTACA |
|  |  |  | Primer R | GGCACTGACATGGGAAGAGT |
|  |  |  | *Probe* | 5’HEX/CAGGACCCA/Zen/TTGCAAGGAACA/3IABkFQ/ |
| Y | MRC | Sry | Primer F | CGTGGTGAGAGGCACAAGTT |
|  |  |  | Primer R | CCAGCTGCTTGCTGATCTC |
|  |  |  | *Probe* | 5’ FAM**™**/CCCAGCAGAATCCCAGCATGCA/BHQ-1 |
| Y | ICS | Sry | Primer F | CATCGGAGGGCTAAAGTGTC |
|  |  |  | Primer R | GTCCCACTGCAGAAGGTTGT |
|  |  |  | *Probe* | 5’6- FAM**™**/GCCTGCAGT/Zen/TGCCTCAACAAA/3IABkFQ/ |

**Supplemental Table ST2: Sequences of primers and probes evaluated for setting up screening panel.**

| Chr | Gene | Genomic Location |  | Sequence (5’-3’) |
| --- | --- | --- | --- | --- |
| 1 | Arhgef4 | 1: 34,678,188-34,813,309 | Primer F | GCACCTAATGGTCTCCTTCCTATG |
|  |  |  | Primer R | CTTAGCAGTCCAGCCTGTTC |
|  |  |  | *Probe* | 5’ FAM**™**/CAGCCCTAACCCAAGGAACTATGTGTC/BHQ-1 |
|  | Klf7 | 1: 64,029,447-64,122,282 | Primer F | TCCAGCACAGGACATGGATT |
|  |  |  | Primer R | CCCTTCCCTTTCCATTAGACTGGTT |
|  |  |  | *Probe* | 5’ FAM**™**/TAGAGGCAAAGCCAACCCCAGC/BHQ-1 |
|  | Kcnj9 | 1: 172,320,501-172,329,318 | Primer F | CCCCTGGTGTTGCCATTTCTAG |
|  |  |  | Primer R | GACGGCTAGGACCTCTTCA |
|  |  |  | *Probe* | 5’ FAM**™**/TCACTCAGGCCTACACTGTCAAGA/BHQ-1 |
| 8 | Primpol | 8: 46,575,579-46,617,212 | Primer F | GGAACTGTTTTAATTCTCTGACTTTCA |
|  |  |  | Primer R | TCCCTGAATTTCATCTCATTGTCTAC |
|  |  |  | *Probe* | 5’ FAM**™**/ACCTGTGGCAAAAGTGAGGTAAAACA/BHQ-1 |
|  | Usp38 | 8: 80,980,734-81,014,906 | Primer F | GGCCATTGGCTCAGCATGT |
|  |  |  | Primer R | TGAACAGCTGCCAGTCCTT |
|  |  |  | *Probe* | 5’ FAM**™**/AGATACGTGGGTAATTGCTCTCCTGA/BHQ-1 |
|  | Gse1 | 8: 120,230,536-120,581,390 | Primer F | ATACGCACCCGGTACTGGAA |
|  |  |  | Primer R | GCAAGCAACGATGAATTTGTGG |
|  |  |  | *Probe* | 5’ FAM**™**/AGGATTAAGGCAACCCTTACATCAGAC/BHQ-1 |
| 11 | Emid1 | 11: 5,106,265-5,152,257 | Primer F | GCCAGGACTGGGTAGCAC |
|  |  |  | Primer R | AGGAGGCTCCTGAATTTGTGACAAG |
|  |  |  | *Probe* | 5’ FAM**™**/CCTGGGTCATCTGAGCTGAGTCC/BHQ-1 |
|  | Kcnj12 | 11: 61,022,564-61,071,131 | Primer F | CACCAGAGTGGGTGCTTCTTAG |
|  |  |  | Primer R | GCCAGACAACCAGAGTCTCTA |
|  |  |  | *Probe* | 5’ FAM™/CCGCTGGACTCACAGCTTGAGTT/BHQ-1 |

All assays were tested for consistency of separation between positive and negative droplets. All assays were tested on temperature gradient and found to the most compatible with the reference assay (*Dot1l*) and with each other i.e. all assays gave consistent results at an annealing temperature of 58ᵒC, thus enabling the screening of all four chromosomes under the same thermal cycling conditions. Assays highlighted in blue are the ones chosen for the screen by MRC Harwell.

**Supplemental Table ST3: ddPCR chromosome counting analysis of ES cells derived of the JM8 parental line**

The table summarises all ddPCR karyotype screening data generated from ES cells, derived from the JM8 parental line that entered our ES cells to mouse conversion process, where four chromosomes were assayed. *Those clones carrying multiple abnormalities are included in more than one category and as such the numbers recorded in the abnormality columns may exceed that recorded in the < 50% euploidy category. Here, euploidy call is based on the chromosomes that were assayed.

|  | **Number of clones (percentage of 378 clones analysed)** |
| --- | --- |
| ≥ **50% euploidy** | 262 (69.3%) |
| < **50% euploidy*** | 116 (30.7%) |
|  |  |
| **ES clones with Chr 1 aneuploidy**** | 10 (2.6%) |
| **ES clones with Chr 8 aneuploidy **** | 86 (22.8%) |
| **ES clones with Chr 11 aneuploidy **** | 22 (5.8%) |
| **ES clones with Chr Y aneuploidy **** | 14 (3.7%) |
|  |  |
| **ES clones with three aneuploid chromosomes (among 1, 8, 11 and Y)** | 1 (0.3%) |
| **ES clones with two aneuploid chromosomes (among 1, 8, 11 and Y)** | 3.2 (10.3%) |
| **ES clones with one aneuploid chromosome among 1, 8, 11 and Y** | 103 (27.2%) |
| **ES clones with only aneuploid Chr 1 only** | 6 (1.6%) |
| **ES clones with only aneuploid Chr 8 only** | 73 (19.3%) |
| **ES clones with only aneuploid Chr 11 only** | 13 (3.4%) |
| **ES clones with only aneuploid Chr Y only** | 10 (2.6%) |

** Only this aneuploidy or in combination with others aneuploidies.

**Supplemental Table ST4: Karyotypic anomalies in non-JM8 derived clones.**

The table summarises the percentage of abnormal clones detected by chromosome counting or ddPCR analysis in JM8 and two other tested ES cell lines. The last column presents the percentage of clone that were not discarded by ddPCR analysis but show more than 50% aneuploid metaphases detected by Giemsa staining metaphase spread-based karyotyping.

| ES cell line | Background | Chromosome counting only* | ddPCR analysis only** | ddPCR analysis followed by chromosome counting*** |
| --- | --- | --- | --- | --- |
| JM8 | C57BL/6N | 27% (715) | 21% (434) | 4% (130) |
| S3 | C57BL/6N | 44% (120) | 24% (264) | 14% (74) |
| TB1 | C57BL/6N | 27% (167) | 8% (104) | 16% (45) |

Number of clones analysed by each method is indicated in brackets.

* Percentage of clones with more than 50% euploid metaphases detected by Giemsa staining metaphase spread-based karyotyping

** Only Chr 8 and Y were analysed

*** Percentage of clone that were not detected abnormal by ddPCR analysis but show more than 50% euploid metaphases detected by Giemsa staining metaphase spread-based karyotyping

**Supplemental Table ST5: Comparison of ddPCR chromosome counting outcome when performed in duplex or multiplex**

The table shows an example of copy counting data obtained on a clone by running assays in duplex (1 FAM™ and 1 VIC® labelled assay at a time) and in multiplex (2 FAM™ and 1 VIC® labelled assay at a time). Note the very similar outcome of duplex and multiplex reactions.

| Assay | Copy Number | Poisson error |
| --- | --- | --- |
| Chr 8 duplex | 2.62 | 0.13 |
| Chr 11 duplex | 1.91 | 0.07 |
| Chr 8 & 11 multiplex both | 4.61 | 0.14 |
| Chr 8 only multiplex | 2.69 | 0.08 |
| Chr 11 only multiplex | 1.92 | 0.06 |
